# Evaluation of serum soluble triggering receptor expressed on myeloid cells-1 in polycystic ovary syndrome

**DOI:** 10.1590/1806-9282.20260286

**Published:** 2026-09-28

**Authors:** Taylan Onat, Senem Arda Duz, Erdinc Saridogan, Selami Gunal, Gamze Nur Cimilli Senocak, Gorkem Tuncay, Abdullah Karaer

**Affiliations:** 1 University of Health Sciences, Sincan Training and Research Hospital – Ankara, Türkiye.; 2 Inonu University, School of Medicine, Department of Obstetrics and Gynecology – Malatya, Türkiye.; 3 Inonu University, School of Pharmacy, Department of Pharmaceutical Microbiology – Malatya, Türkiye.; 4 Ataturk University, School of Medicine, Department of Obstetrics and Gynecology – Erzurum, Türkiye.

**Keywords:** Chronic inflammation, Hyperandrogenism, Insulin resistance, Polycystic ovary syndrome, STREM-1

## Abstract

**OBJECTIVE:**

This study aims to investigate the clinical significance of serum soluble triggering receptor expressed on myeloid cells-1 levels in patients with polycystic ovary syndrome.

**METHODS:**

This case-control study included 40 women with polycystic ovary syndrome and 40 healthy control women. Serum soluble triggering receptor expressed on myeloid cells-1 and high-sensitive C-reactive protein levels were measured via commercially available enzyme-linked immunosorbent assay, according to the manufacturer's instructions.

**RESULTS:**

Soluble triggering receptor expressed on myeloid cells-1 levels were statistically significantly higher in women with polycystic ovary syndrome than controls (p<0.001). In the logistic regression analysis, an increase in serum soluble triggering receptor expressed on myeloid cells-1 level demonstrated a significant association with polycystic ovary syndrome diagnosis (p=0.011). In the receiver operating characteristic curve analysis, the area under the curve (AUC) for soluble triggering receptor expressed on myeloid cells-1 was found to be 0.800 (p<0.001). Setting the cut-off value at 45.22 pg/mL, soluble triggering receptor expressed on myeloid cells-1 demonstrated a sensitivity of 87.5% and a specificity of 65.0%. In the logistic regression analysis that incorporated homeostatic model assessment for insulin resistance, anti-müllerian hormone, and soluble triggering receptor expressed on myeloid cells-1, a 1 pg/mL rise in soluble triggering receptor expressed on myeloid cells-1 was associated with a 1.07-fold increase in the odds of polycystic ovary syndrome, a 3.10-fold increase in the odds due to a 1-point rise in homeostatic model assessment for insulin resistance, and a 3.24-fold increase in the odds for every 1 ng/mL rise in anti-müllerian hormone. The model incorporating anti-müllerian hormone, homeostatic model assessment for insulin resistance, and soluble triggering receptor expressed on myeloid cells-1 predicted polycystic ovary syndrome with 83.8% accuracy, 90.0% specificity, and 77.5% sensitivity.

**CONCLUSION:**

Soluble triggering receptor expressed on myeloid cells-1 may serve as an indicator of the chronic inflammatory process in polycystic ovary syndrome and could be a complementary marker rather than a diagnostic marker for polycystic ovary syndrome.

## INTRODUCTION

Polycystic ovary syndrome (PCOS) is a common endocrinological disorder with varied prevalence depending on the criteria used^1^. The typical clinical presentation frequently includes oligo/amenorrhea, hirsutism, acne, androgenic alopecia, and obesity^2^. The phenotypes of PCOS include a variety of findings and symptoms. It has been found that different phenotypes in PCOS differ in terms of metabolic risks^3^. In addition to the Rotterdam criteria, the National Institutes of Health (NIH) and Androgen Excess-PCOS (AE-PCOS) criteria are also used for diagnosing PCOS. The Rotterdam criteria are used more frequently because they are based on the current recommendations of the 2023 international evidence-based guidelines for the assessment and management of PCOS^2^. Infertility, type 2 diabetes mellitus, dyslipidemia, cardiovascular diseases, cerebrovascular events, and endometrial cancer may occur in the long term^2^. Although PCOS was defined in 1935 and its pathogenesis has been investigated by many studies, its etiopathogenesis remains poorly understood. According to ethnicity and age, the clinical presentation may vary considerably, making PCOS a highly heterogeneous condition^2^. While menstrual irregularities such as oligo/amenorrhea are hallmark features of PCOS, distinguishing these from physiological anovulation during adolescence remains challenging. A recent systematic review and meta-analysis by Zuchelo et al. highlighted the overlap between menstrual patterns in PCOS and hypothalamic–pituitary–ovarian axis immaturity in adolescents, emphasizing the need for careful diagnostic evaluation beyond menstrual history alone^4^.

Evidence suggests that androgens contribute to the inflammatory milieu in patients with PCOS^5^. Furthermore, visceral adipose tissue has been implicated in the chronic inflammatory state observed in patients with PCOS^6^. In addition, several inflammatory markers, including chemokines and cytokines, have been identified in the serum of women with PCOS^5^.

The triggering receptor expressed on myeloid cells 1 (TREM-1) is also associated with the inflammatory process^7^. TREM-1 promotes secretion of interleukin-1β (IL-1β), tumor necrosis factor-α (TNF-α), and monocyte chemotactic protein-1 and initiates the production of some inflammatory cytokines by induction of Toll-like receptor and nucleotide-binding oligomerization domain (NOD)-like receptor pathways^8^. The aim of this study was to investigate the clinical significance of serum soluble triggering receptor expressed on myeloid cells-1 (sTREM-1) levels in patients with PCOS. To the best of our knowledge, this is the first study to evaluate sTREM-1 as an inflammatory marker in patients with PCOS.

## METHODS

This study was designed as a case-control study and was conducted in a tertiary medical centre. The study was performed in accordance with the ethical principles outlined in the Helsinki Declaration. The study period was from July 2022 to January 2023. Atatürk University Faculty of Medicine Ethics Committee for Clinical Research approval was obtained with the number (30.06.2022-6-23). All participants provided written informed consent.

Based on a study conducted by Rudnicka et al.^9^, with a statistical power of 80% and an alpha of 20%, a total of 37 participants per group was needed for the study. We decided on 40 individuals in each group, assuming a dropout rate of 10%. The study included women diagnosed with PCOS aged 18 to 30 years. The diagnosis of PCOS was made using the Rotterdam criteria. Exclusion criteria included the presence of chronic systemic diseases (such as diabetes mellitus), acute infections, and use of oral contraceptives and/or insulin sensitizers within the preceding three months. In addition, the use of medications that affect inflammatory markers (metformin, NSAIDs, etc.) and endocrine disorders such as Cushing's syndrome, congenital adrenal hyperplasia, and thyroid disease were also used as exclusion criteria. Their body mass indexes (BMI), waist–hip ratios (WHR), and Ferriman-Gallwey scores (FGS) were recorded. Fasting venous blood samples were collected during the early follicular phase, following an overnight fast of at least 8 h.

Serum samples acquired during the fasting period from patients with PCOS were obtained within the 2nd–5th day of menstruation and subsequently preserved at −80°C for subsequent analysis. Serum sTREM-1 (Elapscience, Catalog No: E-EL-H1596, USA) and hs-CRP levels were measured using commercially available enzyme-linked immunosorbent assay (ELISA) kits, according to the manufacturer's instructions. After the possibility of pregnancy was ruled out in the group of patients with oligo-amenorrhea, blood samples were collected between the 2nd and 5th days of progesterone-induced menstruation.

Statistical analyses were performed using SPSS Statistics software, version 22. The Kolmogorov-Smirnov test was used to assess the normality of continuous variables. Depending on whether the quantitative data were normally distributed or not, the Student's t-test or Mann-Whitney U test was used. Categorical variables were compared by the chi-square test. To evaluate the associations between numerical data, either the Pearson or Spearman Rho test was employed. The impact of sTREM-1 levels and covariates on PCOS diagnosis was assessed using multivariate binary logistic regression. To avoid multicollinearity, several variables that were significant in the univariate analysis were deliberately excluded from the final model because they were closely related to the covariates that were retained. Specifically, fasting insulin was excluded because it is a component of HOMA-IR, while waist–hip ratio and testosterone were excluded because they closely reflect the metabolic and androgenic status that is already captured by HOMA-IR and AMH. Receiver operating characteristic (ROC) curve analysis was performed to evaluate the predictive performance of sTREM-1 levels for PCOS diagnosis. Statistical significance was defined as p<0.05.

## RESULTS

Table 1 presents the demographic characteristics, biochemical values, and sTREM-1 levels of the study participants. The two groups did not differ significantly with respect to BMI or age. Statistically significant differences were observed in waist-to-hip ratio (WHR) and FGS between the groups (p<0.05). sTREM-1 levels were significantly higher in women with PCOS than in controls (p<0.001). Additionally, total testosterone, free testosterone, thyroid-stimulating hormone (TSH), anti-mullerian hormone (AMH), and hsCRP levels were found to be statistically significant between the two groups (p<0.05) (Table 1). Analysis of the relationships among independent variables revealed a significant positive correlation between sTREM-1 and hsCRP (r=0.471, p<0.001) and AMH (r=0.274, p=0.014), although the latter reflects only a weak association. Additionally, a statistically significant correlation was found between AMH and hsCRP (r=0.251, p=0.024) and Ferriman-Gallwey score (FGS) (r=0.344, p=0.010) (Table 2).

**Table 1:** Clinical and laboratory characteristics of the women with or without polycystic ovary syndrome.

	PCOS (n: 40)	Control (n: 40)	P-value
Age (years)	20.5 (20.5–23)	22 (20–23)	0.100μ
BMI (kg/m^2^)	22.2 (20.1–26.6)	21.2 (19.6–23.3)	0.132μ
WHR	0.797±0.072	0.757±0.068	**0.014** *
FGS	11 (9.75–19.3)	0 (0–5)	**<0.001**μ
HOMA-IR	2.13 (1.67–3.1)	1.48 (1.15–2.0)	**<0.001**μ
AMH (ng/mL)	3.66 (2.88–4.19)	2.5 (1.96–3.08)	**<0.001**μ
FSH (mIU/mL)	5.57±1.31	5.64±1.42	0.817*
LH (mIU/mL)	6.46 (4.04–9.31)	5.61 (4.66–5.99)	0.130μ
Estradiol (pg/mL)	40.2 (31.2–56.0)	40.7 (38.7–51.4)	0.301μ
Prolactin (ng/mL)	14.2 (9.04–19.1)	12.6 (9.73–15.1)	0.229μ
TSH (mIU/L)	2.39 (1.89–3.14)	1.92 (1.49–2.43)	**0.015**μ
fT_4_ (ng/mL)	1.15 (1.02–1.22)	1.08 (0.96–1.16)	0.050μ
DHEA-S (μg/dL)	230 (174–275)	199 (166–231)	0.138μ
Total testosterone (ng/dL)	31.9 (25.1–53.7)	24.0 (20.0–28.8)	**<0.001**μ
Free testosterone (ng/dL)	2.19 (1.84–2.72)	1.83 (1.57–2.06)	**0.004**μ
Fasting glucose (mg/dL)	87.5 (83–91)	86 (81.5–90.5)	0.424μ
Fasting insulin (mIU/mL)	9.66 (7.51–14.8)	6.94 (5.56–9.5)	**0.001**μ
TC (mg/dL)	172 (157–186)	166 (147–179)	0.353μ
LDL-C (mg/dL)	101 (86.5–115)	91.5 (85.5–104)	0.184μ
HDL-C (mg/dL)	52.8±10.8	55.1±10.3	0.340*
TG (mg/dL)	82 (70.8–109)	87 (63.8–107)	0.736μ
hsCRP (mg/L)	2.25±0.887	1.81±0.663	**0.013** *
sTREM-1 (pg/mL)	61.4±21.3	38.2±20.3	**<0.001** *

Data are presented as median (25th–75th percentiles) and mean±standard deviation. The Mann-Whitney U (μ) and Student's t-tests

(*) were used. BMI: body mass index; WHR: waist–hip ratio; FGS: Ferriman-Gallwey score; HOMA-IR: homeostatic model assessment for insulin resistance; AMH: anti-müllerian hormone; FSH: follicle-stimulating hormone; LH: luteinizing hormone; TSH: thyroid-stimulating hormone; fT_4_: free thyroxine; DHEA-S: dehydroepiandrosterone sulfate; TC: total cholesterol; LDL-C: low-density lipoprotein cholesterol; HDL-C: high-density lipoprotein cholesterol; TG: triglyceride; hsCRP: high-sensitive C-reactive protein; sTREM-1: soluble form of triggering receptor expressed on myeloid cells; PCOS: polycystic ovary syndrome. Bold values indicate statistical significance (p<0.05).

**Table 2:** The relationships between independent variables.

		sTREM-1	HOMA-IR	WHR	FGS	Total testosterone	hsCRP
HOMA-IR	Correlation coefficient	0.143	–				
	p-value	0.206	–				
WHR	Correlation coefficient	0.122	0.130	–			
	p-value	0.281	0.250	–			
FGS	Correlation coefficient	0.289	0.420	0.180	–		
	p-value	**0.009**	**<0.001**	0.109	–		
Total testosterone	Correlation coefficient	0.147	0.027	0.215	0.350	–	
	p-value	0.194	0.814	0.055	**0.001**	–	
hsCRP	Correlation coefficient	0.373	0.021	0.183	0.159	0.313	–
	p-value	**<0.001**	0.856	0.104	0.160	**0.005**	–
AMH	Correlation coefficient	0.274	0.135	0.084	0.186	0.199	0.251
	p-value	**0.014**	0.233	0.458	0.099	0.077	**0.024**

Correlation analyses of independent variables were performed using Spearman's Rho tests. sTREM-1: soluble form of triggering receptor expressed on myeloid cells; HOMA-IR: homeostatic model assessment for insulin resistance; WHR: waist–hip ratio; FGS: Ferriman-Gallwey score; hsCRP: high-sensitive C-reactive protein; AMH: anti-müllerian hormone. Bold values indicate statistical significance (p<0.05).

ROC curve analysis demonstrated AUC values of 0.800 for sTREM-1 and 0.785 for AMH (Figure 1). Setting the cut-off value at 2.81 ng/mL, AMH demonstrated sensitivity of 77.5%, specificity of 70.0%, and accuracy of 73.75%. For sTREM-1, at the cut-off value determined as 45.22 pg/mL, specificity was detected to be 65.0%, sensitivity 87.5%, and accuracy 76.25%.

**Figure 1:**
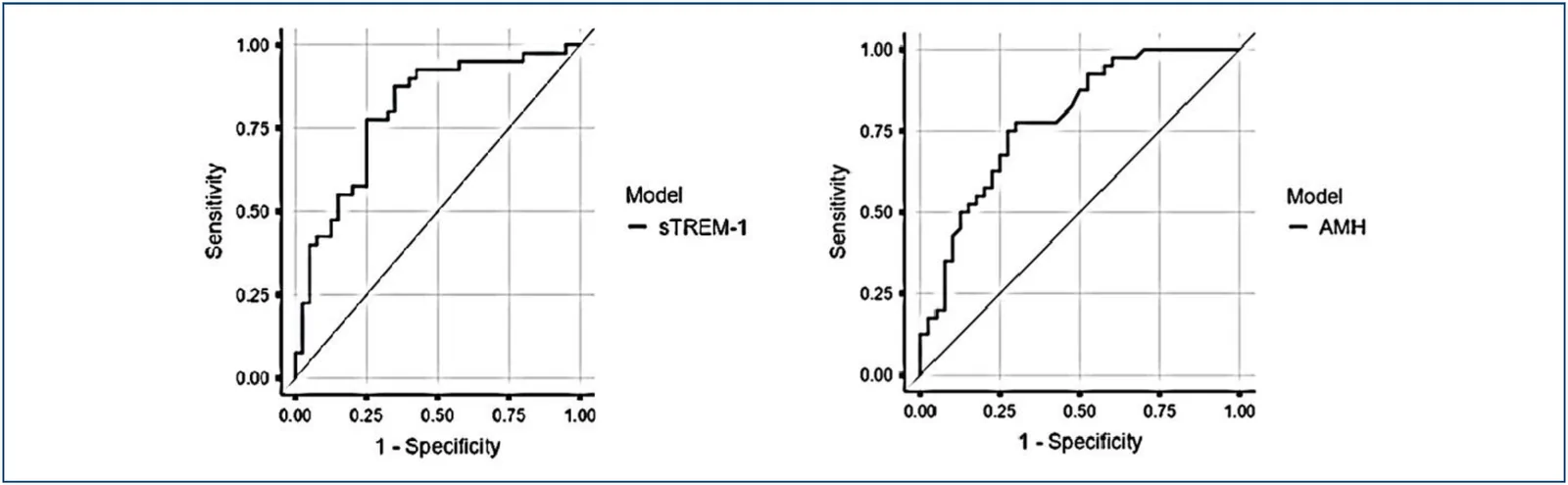
Receiver operating characteristiccurve analysis of soluble triggering receptor expressed on myeloid cells-1 and anti-müllerian hormone.

In the logistic regression analysis that incorporated HOMA-IR, AMH, and sTREM-1, a 1 pg/mL rise in sTREM-1 was associated with a 1.07-fold increase in the odds of PCOS, a 3.10-fold increase in the odds due to a 1-point rise in HOMA-IR, and a 3.24-fold increase in the odds for every 1 ng/mL rise in AMH. The model incorporating AMH, HOMA-IR, and sTREM-1 predicted PCOS with 83.8% accuracy, 90.0% specificity, and 77.5% sensitivity.

## DISCUSSION

This study is the first to demonstrate an association between sTREM-1 levels and PCOS. The fact that sTREM-1 shows a moderate positive correlation with hsCRP supports the hypothesis that sTREM-1 may serve as a marker of chronic low-grade inflammation, which plays a role in the pathogenesis of PCOS. Notably, the PCOS group exhibited significantly higher levels of sTREM-1 and AMH. Furthermore, sTREM-1, AMH, and HOMA-IR were identified as factors significantly associated with PCOS.

The relationship between AMH, IR, and inflammation plays a significant role in the pathophysiology of PCOS. IR and the resulting hyperinsulinemia are considered key drivers of PCOS pathophysiology, contributing to hyperandrogenism and reproductive dysfunction^10^. Unlike other target organs, such as the liver and skeletal muscle, the ovaries maintain insulin sensitivity through the phosphatidylinositol 3-kinase-independent insulin signaling pathway^11^. Insulin-induced increased androgen synthesis by theca cells increases the number of pre-antral follicles, which causes a rise in AMH, disrupting follicular maturation. Elevated AMH, in conjunction with insulin resistance, has been demonstrated to suppress the conversion of androgens to estrogen in granulosa cells by inducing aromatase suppression. Tumor necrosis factor alpha (TNF-α) can cause insulin resistance by inhibiting insulin receptor tyrosine kinase in target organs^12^. There are studies suggesting that TREM1 may regulate the release of proinflammatory cytokines such as TNF-α and IL-8^13^. The relationship between hyperandrogenism, insulin resistance (IR), and chronic low-grade inflammation in PCOS is well-established. In this study, significant differences were observed between the two groups in the clinical and biochemical findings of hyperandrogenism, HOMA-IR values, and inflammation markers. However, no correlation was found among these parameters. The potential causes of this phenomenon may include the heterogeneous nature of PCOS and the limited sample size of the study. Alternatively, sTREM-1 may reflect a systemic inflammatory state that is partly independent of the metabolic and androgenic features of PCOS, or it may be influenced by unmeasured factors such as adiposity, dietary habits, and physical activity, which were not assessed in the present study.

Diagnosing PCOS during adolescence is challenging because oligo-amenorrhea is frequently observed during this period, as the hypothalamic–pituitary axis has not yet fully matured. There is currently no consensus on this issue. In their meta-analysis, Zuchelo et al. found that adolescents with PCOS had a higher BMI compared to adolescents with oligo-amenorrhea and that amenorrhea is a significant finding for PCOS^4^. The results of our study support the notion that sTREM could be a candidate marker for use as a complementary marker in suspected cases.

Inflammation is a normal immunological response to injury or infection; however, when persistent, it may cause tissue damage and contribute to the development of chronic conditions such as PCOS. Recent evidence suggests that PCOS may be related to chronic low-grade inflammation^14^. Inflammation is a diverse biological response that is activated in response to cellular injury and is involved in many physiological processes. The chronic inflammatory state observed in PCOS may have implications beyond metabolic and reproductive dysfunction. Baracat et al. demonstrated that hormonal and metabolic factors influence progesterone action on the endometrium in women with PCOS, potentially contributing to the endometrial dysfunction and increased cancer risk associated with this condition^15^. While our study focused on sTREM-1 as a marker of systemic inflammation, future research should explore whether elevated sTREM-1 levels correlate with endometrial inflammatory markers and whether this contributes to the endometrial abnormalities observed in PCOS. One of the theories is that the ovaries of women with PCOS are subject to chronic low-level oxidative stress, which can trigger an inflammatory response^16^. Oxidative stress is thought to result from factors such as insulin resistance and elevated androgen levels, which are commonly observed in women with PCOS^16^.

Elevated hs-CRP concentrations have been associated with increased cardiovascular risk, which is of particular concern in women with PCOS, who are already at elevated risk for cardiovascular disease^17^. Several studies have investigated the role of hs-CRP in PCOS^18^. These studies have found that women with PCOS have higher levels of hs-CRP than controls^18^. Furthermore, research suggests that inflammatory markers may not be raised in women with non-obese PCOS^19^. These discrepancies may be attributed to differences in study design, patient populations, and other methodological factors.

AMH is a member of the transforming growth factor beta family, which has been used to assess ovarian reserve until recent years. However, its diagnostic performance, particularly in PCOS, has now begun to be included in guidelines^2^. When women with ovulatory cycles are compared to women with PCOS, it has been found that women with PCOS have higher AMH levels. Barghi et al.^20^ discovered in their meta-analysis that AMH levels are elevated in women with PCOS and diminish with advancing age and increasing BMI. In the study, we used two groups matched for age and BMI to isolate the effects of these factors on AMH. The results of our study align with existing literature on the subject, which has demonstrated that AMH levels are elevated in individuals diagnosed with PCOS.

sTREM-1, a biomarker of inflammation, plays a crucial role in triggering the innate immune response. This cell surface molecule is expressed by both neutrophils and monocytes, which are actively involved in fueling the propagation of the inflammatory response^21^. TREM-1 levels are influenced by both infectious and inflammatory diseases^21-24^. Evidence indicates that sTREM-1 promotes the generation of pro-inflammatory cytokines, including TNF-α^22^. Ferat-Osorio et al. noted that sTREM-1 reflects an inflammatory state rather than an infectious one^23^. TREM-1 expression was shown to be greater in diabetes-obese individuals compared to non-diabetic obese patients, implying a link between insulin resistance and TREM-1^24^. Notably, sTREM-1 represents a promising therapeutic target for pharmacological intervention^22^. In this study, sTREM-1 levels were found to be significantly higher in women with PCOS. Notably, this difference persisted despite the PCOS and control groups being matched for BMI, suggesting that the elevation in sTREM-1 reflects an intrinsic PCOS-related inflammatory component rather than merely an effect of adiposity.

Although insulin resistance is not a diagnostic criterion for PCOS, it is frequently observed even in lean women with PCOS. Lim et al. conducted a meta-analysis that revealed the detrimental effect of obesity on metabolic and reproductive outcomes in women with PCOS^25^. Guo et al. found that the clinical and biochemical signs of PCOS have been altered with age^26^. According to that study, symptoms of hyperandrogenism predominate at younger ages, while metabolic symptoms become more prominent with advancing age^26^.

There are some limitations of this study. Because this study is a case-control study, a cause-and-effect relationship could not be established. Additional limitations include the small sample size, the inability to stratify participants according to PCOS subgroups, and the use of a single blood sample per participant. In addition, factors such as excess weight, physical activity, and dietary habits, which we did not assess in this study, represent a significant limitation. Different PCOS phenotypes exhibit varying degrees of metabolic dysfunction and inflammatory profiles. Soares-Jr et al. demonstrated that PCOS phenotypes influence the development of metabolic syndrome over a six-year follow-up, with phenotype A (hyperandrogenism+oligo/amenorrhea+polycystic ovary morphology) showing the highest metabolic risk^3^. The small sample size may have limited statistical power to detect modest correlations between sTREM-1 and metabolic parameters such as HOMA-IR or clinical markers of hyperandrogenism (FGS); these null findings should therefore be interpreted with caution and do not preclude a true association. On the other hand, this study is the first to examine the relationship between PCOS and sTREM-1.

In conclusion, sTREM-1 appears to be associated with the chronic inflammatory process in PCOS and may contribute to our understanding of the role of inflammation in its pathogenesis. sTREM-1 may serve as an indicator of the chronic inflammatory process in PCOS and could be a complementary marker rather than a diagnostic marker for PCOS. Given the heterogeneous nature of PCOS, these findings need to be validated by studies conducted in larger, well-defined cohorts that include both lean and obese women from different PCOS subgroups. In addition, future studies could evaluate sTREM-1 levels in adolescents and in populations receiving PCOS treatment.

## Data Availability

The datasets generated and/or analyzed during the current study are available from the corresponding author upon reasonable request.
